# Anti‐Müllerian Hormone and Metabolic–Hormonal Profiles in Women With and Without Polycystic Ovary Syndrome: A Population‐Based Study

**DOI:** 10.1002/edm2.70287

**Published:** 2026-07-30

**Authors:** Fahimeh Ramezani Tehrani, Maryam Mousavi, Marzieh Saei Ghare Naz, Maryam Farahmand, Masoumeh Jorjani, Fatemeh Mahboobifard

**Affiliations:** ^1^ Reproductive Endocrinology Research Center, Research Institute for Endocrine Molecular Biology, Research Institute for Endocrine Sciences Shahid Beheshti University of Medical Sciences Tehran Iran; ^2^ Foundation for Research & Education Excellence Vestavia Hills Alabama USA; ^3^ Department of Pharmacology, School of Medicine & Neurobiology Research Center Institute of Neuroscience and Cognition, Shahid Beheshti University of Medical Sciences Tehran Iran; ^4^ Department of Pharmacology School of Medicine, Shahid Beheshti University of Medical Sciences Tehran Iran

**Keywords:** Anti‐Müllerian hormone, Metabolic profile, Polycystic ovary syndrome

## Abstract

**Introduction:**

Evidence regarding age‐related variation in anti‐Müllerian hormone (AMH) and its associations with hormonal and metabolic parameters in women with polycystic ovary syndrome (PCOS), healthy women, and those with isolated PCOS features remains inconsistent. This study aimed to address these gaps using a cross‐sectional, population‐based approach.

**Methods:**

A total of 883 women aged 18–45 years were categorized into three groups: PCOS (*n* = 124; Rotterdam criteria), suspicious (one PCOS criterion, *n* = 274), and healthy controls (*n* = 485). The coefficient of determination (R^2^) assessed age‐related AMH variation. Linear regression evaluated associations between AMH and hormonal and metabolic profiles. Age‐stratified multivariable logistic regression, adjusted for BMI, parity, and physical activity, examined associations between AMH and type 2 diabetes (T2DM), hypertension, metabolic syndrome (MetS), and dyslipidemia.

**Results:**

Age explained nearly 40% of AMH variation in healthy women, but only 20% in those with PCOS. AMH was inversely correlated with BMI in healthy and suspicious groups, but not in PCOS. Among healthy women aged ≥ 35 years, higher AMH was associated with a 37% reduced likelihood of T2DM (OR: 0.63, 95% CI: 0.41–0.97). In contrast, among women with PCOS aged < 35 years, elevated AMH was associated with 2.6‐fold higher odds of MetS (OR: 2.6, 95% CI: 1.20–6.20). In the suspicious group, AMH correlated positively with free androgen index and prolactin.

**Conclusion:**

AMH declines less with age in women with PCOS than in healthy women. Higher AMH may be protective against T2DM in older healthy women but indicates elevated metabolic risk in younger women with PCOS, suggesting that AMH may serve as a life‐course biomarker for metabolic risk stratification and personalized preventive strategies in women's healthcare.

## Graphical abstract

In this population‐based study of 883 women, AMH declined less with age in women with PCOS than in healthy women. Higher AMH may protect older healthy women from T2DM but indicates elevated metabolic risk in younger women with PCOS, supporting its role as a life‐course biomarker for metabolic risk stratification.

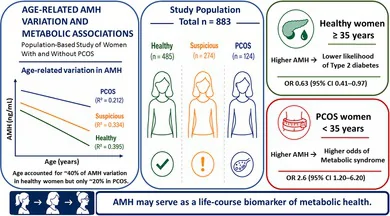

## 1 Introduction

Polycystic ovary syndrome (PCOS) is the most common endocrine disorder among women of reproductive age, affecting approximately 5%–20% of this population [1]. Although formal diagnostic criteria for PCOS were first established during the National Institutes of Health (NIH) conference in 1990, these criteria have evolved over time, reflecting ongoing uncertainties and gaps in understanding the syndrome's aetiology and pathophysiology [2]. Furthermore, the underlying mechanisms of PCOS remain incompletely understood, posing challenges for both diagnosis and management. Therefore, further research is needed to elucidate the pathogenesis of PCOS and improve diagnostic and therapeutic strategies [3].

Anti‐Müllerian hormone (AMH), a well‐established marker of ovarian reserve, is secreted by granulosa cells of preantral and small antral follicles. Women with PCOS exhibit markedly elevated AMH levels owing to the increased number of small follicles characteristic of the disorder [2]. Multiple molecular and hormonal mechanisms contribute to this elevation. Impaired follicle‐stimulating hormone (FSH) signalling leads to follicular arrest and the accumulation of AMH‐producing follicles, whereas hyperandrogenism, elevated luteinizing hormone (LH) levels, and insulin resistance promote granulosa cell proliferation and AMH secretion [4]. In addition, dysregulated transforming growth factor‐*β* (TGF‐*β*) signalling may further increase AMH expression, which in turn suppresses FSH activity, perpetuating disrupted folliculogenesis and sustained AMH overproduction in PCOS [5, 6].

It is well established that AMH levels decline with age in both healthy women and those with PCOS, although this decline appears to occur more gradually in women with PCOS [7, 8]. Nevertheless, the extent to which age contributes to AMH variation within each group, and whether age‐related patterns differ significantly between women with and without PCOS, remains unclear. Furthermore, the association between body mass index (BMI) and AMH levels in healthy women with regular menstrual cycles remains inconsistent [9], and comparable data in women with PCOS are limited, particularly in unselected population‐based cohorts.

The interplay between AMH and hormonal and metabolic profiles, as well as its potential influence on metabolic outcomes such as type 2 diabetes mellitus (T2DM), hypertension (HTN), metabolic syndrome (MetS), and dyslipidemia, remains poorly characterized in both women with PCOS and healthy women. The use of a population‐based sample enables the evaluation of AMH across a broader spectrum of reproductive and metabolic phenotypes. In contrast, clinic‐based studies often underrepresent milder forms of PCOS, which comprise the majority of cases. Furthermore, women who meet only a single diagnostic criterion for PCOS may represent an at‐risk group whose hormonal and metabolic characteristics remain largely unexplored. Studying women with isolated PCOS features may provide valuable insights into early pathophysiological alterations, potential progression towards full PCOS, and markers associated with future metabolic risk.

## 2 Methods

### 2.1 Study Population

Participants were selected from the Tehran Lipid and Glucose Study (TLGS), a population‐based, multicenter study initiated in 1998. This ongoing study includes a geographically defined population of 15,005 individuals from Tehran, Iran, and aims to assess the incidence and prevalence of metabolic disorders and their associated risk factors for non‐communicable diseases (NCDs). Detailed descriptions of the TLGS have been published previously [10].

All participants provided written informed consent. The study protocol was conducted in accordance with the Declaration of Helsinki and was approved by the Ethics Committee of Shahid Beheshti University of Medical Sciences (Ethics Code: IR.SBMU.MSP.REC.1402.65). The study was designed and reported following the STROBE guidelines for observational research.

Data for the current study were derived from the third follow‐up phase of the TLGS (2005–2008). In addition to the general TLGS questionnaire, participants completed a reproductive history questionnaire covering menstrual characteristics, gynecologic history, hyperandrogenic symptoms, and family history of irregular menstrual cycles and hirsutism. Data were collected through face‐to‐face interviews with women aged 18 to 45 years (*n* = 1960). Clinical assessments included measurements of body weight, height, waist circumference (WC), hip circumference (HC), blood pressure, and assessment of hirsutism and acne severity [11]. Fasting blood samples were collected during cycle days 2–3 (spontaneous or progesterone‐induced cycles) and stored at −80°C until analysis. Participants underwent ovarian ultrasonography, performed transabdominally in virgins and transvaginally in non‐virgins using 3.5‐ and 5‐MHz transducers, respectively. All ultrasonographic examinations were performed on the same day as blood collection.

**FIGURE 1:**
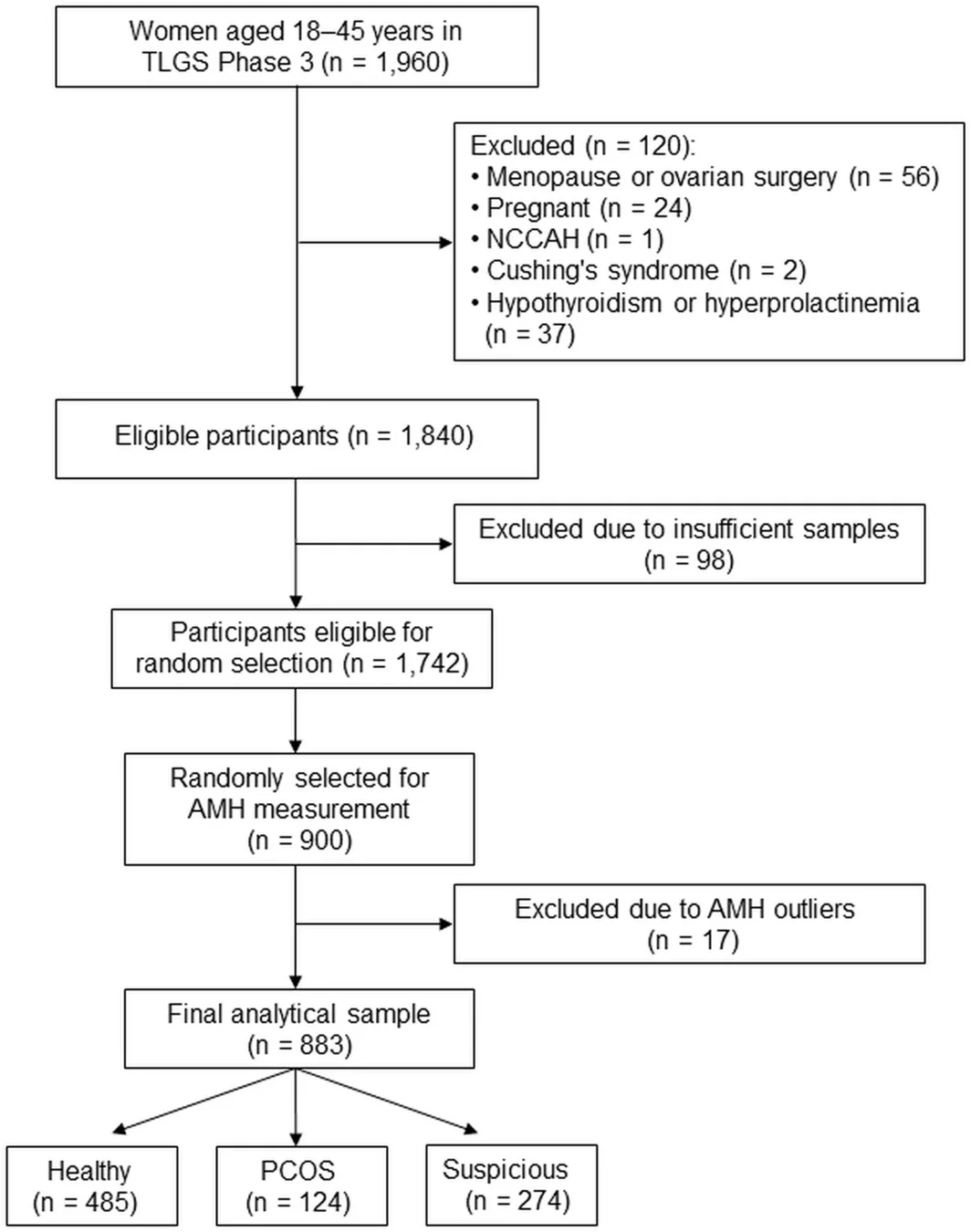
Flow diagram of participant selection and classification. Abbreviations: AMH, anti‐Müllerian hormone; NCCAH, non‐classical congenital adrenal hyperplasia; PCOS, polycystic ovary syndrome; TLGS, Tehran Lipid and Glucose Study.

For the current study, we excluded women with natural or surgical menopause or a history of ovarian surgery (*n* = 56), pregnant women at the time of assessment (*n* = 24), and women with endocrine disorders, including non‐classical congenital adrenal hyperplasia (NCCAH) due to 21‐hydroxylase deficiency (*n* = 1), Cushing's syndrome (*n* = 2), hypothyroidism, or hyperprolactinemia (*n* = 37). Additionally, women with insufficient stored blood samples for AMH measurement were excluded (*n* = 98). From the remaining 1742 eligible participants, 900 women were randomly selected for AMH assessment due to budgetary limitations. Finally, 17 participants were excluded due to outlier AMH values, leaving 883 women in the final analytical sample. The participant selection and classification process is summarized in Figure 1.

Participants were classified into three groups: control, suspicious, and PCOS, based on their status relative to established PCOS criteria. PCOS was defined according to the Rotterdam criteria, requiring the presence of at least two of the following: (1) oligo/anovulation (ANOVU), (2) hyperandrogenemia and/or hyperandrogenism (HA), and (3) polycystic ovarian morphology (PCOM). Women exhibiting only one of these criteria (i.e., ANOVU, HA, or PCOM) were categorized as the suspicious group. Participants who did not meet any of the criteria were classified as the control group.

### 2.2 Definition of Terms

Oligo/anovulation was defined as menstrual cycles ≥ 35 days or a history of fewer than eight menstrual cycles per year [12, 13]. Clinical hyperandrogenism was defined as the presence of hirsutism (modified Ferriman–Gallwey score ≥ 8) [11], acne, or androgenic alopecia. Acne severity was assessed quantitatively based on lesion type, number, and distribution [14].

Biochemical hyperandrogenism was defined as serum dehydroepiandrosterone sulphate (DHEAS), testosterone, or androstenedione (A4) concentrations above the 95th percentile of healthy, non‐hirsute, eumenorrheic women [15]. PCOM was defined according to the Rotterdam criteria as the presence of ≥ 12 follicles measuring 2–9 mm in diameter in at least one ovary and/or an ovarian volume > 10 cm^3^ [16].

Body mass index (BMI) was calculated as weight (kg) divided by height squared (m^2^). Waist circumference (WC) was measured midway between the lower costal margin and the iliac crest, and hip circumference (HC) at the level of the anterior superior iliac spine. Waist‐to‐hip ratio (WHR) was calculated as WC divided by HC, with both measurements recorded in centimetres [17].

MetS was defined according to the Joint Interim Statement criteria [18] as the presence of three or more of the following components: (1) fasting triglycerides (TG) ≥ 150 mg/dL; (2) fasting high‐density lipoprotein cholesterol (HDL‐C) ≤ 50 mg/dL; (3) systolic blood pressure (SBP) ≥ 130 mmHg or diastolic blood pressure (DBP) ≥ 85 mmHg; (4) fasting blood sugar (FBS) ≥ 100 mg/dL; (5) WC > 90 cm, based on population‐ and country‐specific cutoff points for Iranians [19].

T2DM was defined according to the American Diabetes Association's criteria: a 2‐h post‐challenge plasma glucose (2‐h PCPG) ≥ 11.1 mmol/L, fasting plasma glucose (FPG) ≥ 7 mmol/L, or the use of any anti‐diabetic medication [20].

Dyslipidemia was defined as: Serum total cholesterol (TC) ≥ 6.19 mmol/L; Triglycerides (TGs) ≥ 2.26 mmol/L; HDL‐C < 1.036 mmol/L; non‐HDL‐C ≥ 5.15 mmol/L; TC/HDL‐C ≥ 5.97; TG/HDL‐C ≥ 2.18 [21].

### 2.3 Laboratory Measurements

Details of the measurements of FBS and lipid profile parameters have been reported previously [17]. DHEAS, 17‐hydroxyprogesterone (17OH‐P), total testosterone (TT), and A4 levels were measured by enzyme immunoassay (Diagnostic Biochem Canada, Ontario). Sex Hormone Binding Globulin (SHBG) was measured by immunoenzymometric assay (Mercodia, Sweden). All ELISA tests were conducted using a Sunrise ELISA reader (Tecan Co., Salzburg, Austria). LH, FSH, prolactin (PRL), and thyroid‐stimulating hormone (TSH) were measured using an immunoradiometric assay (IRMA) from Izotop, Budapest, Hungary, with a Wallac Wizard gamma counter from Turku, Finland. AMH was measured with ELISA (Gen II Kit, Beckman Coulter, USA) on a Sunrise reader (Tecan Co., Salzburg, Austria). The AMH Gen II control (A79766) was applied at two different concentrations to assess the accuracy of the assays.

The intra‐ and inter‐assay coefficients of variation were as follows: for TT, 5.6% and 6.6%; for DHEAS, 2.0% and 5.1%; for 17 OH‐P, 4.8% and 6.8%; for SHBG, 1.2% and 5.7%; for A4, 2.2% and 3.5%; for LH, 3% and 5.8%; for FSH, 3.5% and 4%; for TSH, 1.7% and 3.4%; for PRL, 2.1% and 4.1%; and for AMH, 1.9% and 2.0%.

The free androgen index (FAI) was calculated as (TT/SHBG) × 100.

### 2.4 Statistical Analysis

Data are presented as mean ± standard deviation (SD) for normally distributed variables, median (interquartile range, IQR) for skewed variables, and number (percentage) for categorical data. Comparisons among the PCOS, suspicious, and control groups were performed using one‐way analysis of variance (ANOVA) for normally distributed variables, the Kruskal–Wallis test for skewed variables, and the chi‐square test for categorical variables.

Median predicted AMH levels were compared across groups after adjustment for age and BMI. Scatter plots were used to illustrate the relationship between AMH and age and BMI.

The coefficient of determination (R^2^) was used to quantify the proportion of variation in AMH levels explained by age within the PCOS, suspicious, and control groups. Multivariable linear regression analyses, adjusted for age and BMI, were performed to evaluate associations between AMH levels and hormonal and metabolic parameters.

To investigate the associations between AMH levels and metabolic outcomes (T2DM, HTN, MetS, and dyslipidemia), age‐stratified multivariable logistic regression was performed to minimize potential confounding by biological aging. All models were further adjusted for BMI, parity, and physical activity. A two‐sided *p* value < 0.05 was considered statistically significant.

## 3 Results

Of the 883 women included in the analysis, 124 (14%) had PCOS, 485 (54.9%) were healthy, and 274 (31%) were categorized as suspicious. The clinical and endocrine characteristics of the three groups are summarized in Table 1. Compared with healthy and suspicious women, those with PCOS were younger (33.62 ± 6.6 vs. 35.43 ± 6.94 and 35.59 ± 6.11 years, respectively; *p* = 0.008), had higher AMH levels [2.21 (IQR: 0.85–3.38) vs. 1.21 (IQR: 0.39–2.62) and 1.19 (IQR: 0.50–2.63) ng/mL; *p* < 0.001], and higher BMI (27.55 ± 5.4 vs. 26.41 ± 4.5 and 26.84 ± 4.7 kg/m^2^; *p* = 0.057). No significant differences were observed in metabolic parameters, including FBS, TC, HDL, and LDL, except for TG, which was significantly higher in the PCOS group (*p* = 0.025).

**TABLE 1:** Clinical and endocrine characteristics of healthy, PCOS, and suspicious women.

	Healthy (*n* = 485)	PCOS (*n* = 124)	Suspicious (*n* = 274)	*p*
Age (years)	35.43 ± 7.10	33.62 ± 6.60	35.59 ± 6.11	**0.008** ^⁎^
WC (cm)	84.36 ± 10.66	84.86 ± 11.60	84.19 ± 10.54	0.907
HC (cm)	102.78 ± 8.40	104.61 ± 9.85	103.98 ± 8.81	0.115
WHR	0.81 ± 0.06	0.80 ± 0.06	0.80 ± 0.07	0.130
WrC (cm)	15.76 ± 0.91	15.88 ± 1.13	15.83 ± 0.92	0.456
BMI (kg/m^2^)	26.41 ± 4.5	27.55 ± 5.4	26.84 ± 4.7	0.057
AMH (ng/mL)^†^	1.21 (0.39–2.62)	2.21 (0.85–3.38)	1.19 (0.50–2.63)	**< 0.001** ^⁎^
TG (mmol/L)^†^	104.50 (77–153.75)	116 (91–161.25)	113 (81.75–156.25)	**0.025** ^⁎^
FBS (mg/dL)	89.71 ± 20.71	88.53 ± 10	89.68 ± 21.14	0.509
TC (mmol/L)	191.23 ± 33.6	192.24 ± 39.40	193.72 ± 33.9	0.639
HDL (mmol/L)^†^	42 (39–49)	42 (35–49)	42 (35–49)	0.230
LDL (mmol/L)	122.6 ± 30	121.91 ± 31.23	124.32 ± 30.41	0.687
LH/FSH^†^	0.58 (0.39–0.76)	0.82 (0.58–1.23)	0.53 (0.37–0.82)	**< 0.001** ^⁎^
TT (nmol/L)^†^	0.35 (0.24–0.65)	0.45 (0.29–0.78)	0.30 (0.14–0.46)	**< 0.001** ^⁎^
DHEAS (mcg/dL)	138.50 ± 72.11	155.05 ± 88.91	150.40 ± 90.46	0.420
SHBG (nmol/L)^†^	60.85 (47.22–79.85)	42.80 (29.64–56.70)	47.90 (29.2–72.7)	**< 0.001** ^⁎^
FAI	0.80 (0.41–1.09)	1.12 (0.53–2.12)	0.64 (0.33–1.07)	**< 0.001** ^⁎^

*Note:* Continuous variables are presented as mean ± standard deviation (SD) or median (interquartile range, IQR), as appropriate.

Abbreviations: AMH, anti‐Müllerian hormone; BMI, body mass index; DHEAS, dehydroepiandrosterone sulphate; FAI, free androgen index; FBS, fasting blood sugar; FSH, follicle‐stimulating hormone; HC, hip circumference; HDL, high‐density lipoprotein; LDL, low‐density lipoprotein; LH, luteinizing hormone; SHBG, sex hormone‐binding globulin; TC, total cholesterol; TG, triglycerides; TT, total testosterone; WC, waist circumference; WHR, waist‐to‐hip ratio; WrC, wrist circumference.

^**⁎**^ Statistically significant (*p* < 0.05).

^**†**^ Variable reported as median (25th–75th percentile, IQR).

After adjustment for age and BMI, the median predicted AMH levels were 2.09 ± 1.03 ng/mL in the PCOS group, 1.80 ± 1.20 ng/mL in the healthy group, and 1.82 ± 1.03 ng/mL in the suspicious group (*p* = 0.031).

**FIGURE 2:**
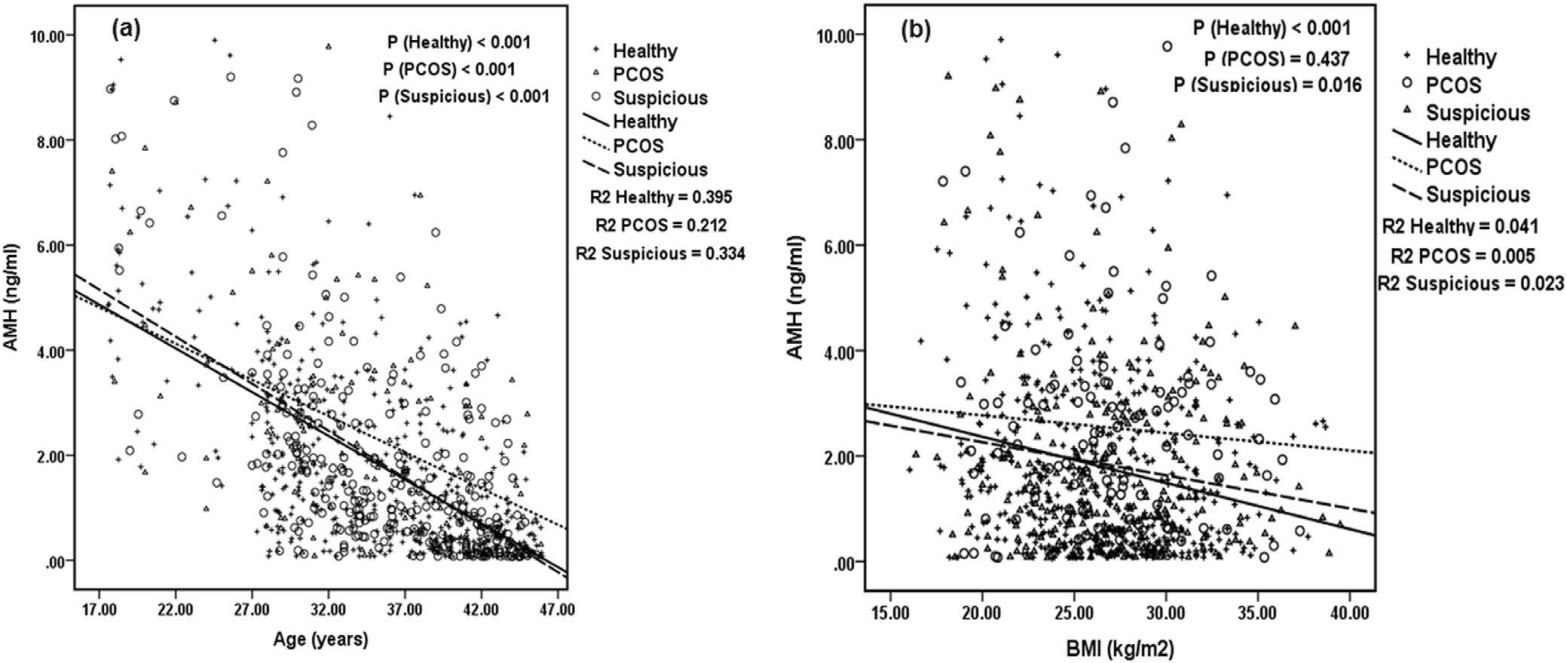
Scatter plot matrix of AMH levels versus (a) age and (b) BMI across the PCOS, healthy, and suspicious groups. Abbreviations: AMH, anti‐Müllerian hormone; BMI, body mass index; PCOS, polycystic ovary syndrome.

As shown in Figure 2a, AMH levels declined significantly with increasing age in all three groups (*p* < 0.001), with the strongest inverse correlation in healthy women (r = −0.63), followed by suspicious (r = −0.58) and PCOS (r = −0.46) groups.

The coefficient of determination (R^2^) for age explaining AMH variance was 0.212 in PCOS, 0.395 in healthy, and 0.334 in suspicious women, nearly double in healthy compared with PCOS participants.

Figure 2b shows inverse correlations between BMI and AMH levels in the healthy (r = −0.20, *p* < 0.001) and suspicious (r = −0.15, *p* = 0.016) groups, whereas no significant correlation was observed in the PCOS group (r = −0.07, *p* = 0.437).

Linear regression models adjusted for age and BMI were used to assess associations between AMH levels and hormonal (TT, SHBG, FAI, DHEAS, A4, LH, FSH, TSH, and PRL) and metabolic (FBS, TC, TG, HDL, and LDL) parameters in the healthy, PCOS, and suspicious groups (Table 2).

**TABLE 2:** Beta coefficients from linear regression assessing the effect of AMH on hormonal and metabolic variables.

Dependent variable	Group‐specific *β* coefficients		
Hormonal profile	Healthy *β* (95% CI)	PCOS *β* (95% CI)	Suspicious *β* (95% CI)
TT	0.00 (−0.04–0.04)	0.01 (−0.06–0.07)	**0.03 (0.01–0.06)**
SHBG	0.07 (−3.78–3.65)	−1.31 (−5.31–2.69)	0.91 (−2.52–4.34)
FAI	0.02 (−0.36–0.49)	0.08 (−0.21–0.23)	**0.18 (0.13–0.95)**
DHEAS	0.85 (−10.22–11.91)	−7.27 (−18.21–3.67)	−2.74 (−15.72–10.23)
A4	−1.13 (−3.32–1.06)	−0.03 (−0.16–0.09)	0.07 (−0.03–0.16)
LH	−0.69 (−1.51–0.13)	−0.75 (−1.57–0.07)	0.44 (−0.61–1.49)
FSH	−0.25 (−1.45–0.94)	0.33 (−0.39–1.05)	−0.58 (−1.98–0.81)
TSH	−0.05 (−0.45–0.36)	0.05 (−0.33–0.43)	−0.02 (−0.28–0.24)
PRL	0.36 (−6.19–6.90)	8.37 (−10.05–26.80)	**14.68 (6.01–23.35)**
Metabolic profile			
FBS	0.04 (−1.32–1.39)	−0.39 (−1.41–0.63)	1.27 (−0.45–2.99)
TC	−1.07 (−3.14–1.00)	0.65 (−3.38–4.68)	−0.17 (−2.83–2.49)
TG	−0.25 (−4.27–3.76)	4.05 (−3.00–11.10)	0.93 (−4.13–5.97)
HDL	−0.45 (−1.10–0.21)	−0.87 (−1.94–0.20)	0.08 (−0.76–0.92)
LDL	−0.66 (−2.54–1.22)	−0.51 (−3.15–4.16)	−0.47 (−2.90–1.96)

*Note:* All models were adjusted for age and BMI. Significant associations (*p* < 0.05) are shown in bold.

Abbreviations: A4, androstenedione; AMH, anti‐Müllerian hormone; DHEAS, dehydroepiandrosterone sulphate; FAI, free androgen index; FBS, fasting blood sugar; FSH, follicle‐stimulating hormone; HDL, high‐density lipoprotein; LDL, low‐density lipoprotein; LH, luteinizing hormone; PRL, prolactin; SHBG, sex hormone‐binding globulin; TC, total cholesterol; TG, triglycerides; TSH, thyroid‐stimulating hormone; TT, total testosterone.

No significant associations were observed in either the healthy or PCOS groups. However, in the suspicious group, AMH showed a weak positive association with TT (*β* = 0.03, 95% CI: 0.01–0.06, *p* = 0.02) and a modest positive association with FAI (*β* = 0.18, 95% CI: 0.13–0.95, *p* = 0.01), indicating that each 1‐unit increase in AMH corresponded to an approximately 0.2‐unit rise in FAI. Furthermore, it was estimated that for each 1‐unit increase in AMH in the suspicious group, the PRL level increased by approximately 15 units (*β* = 14.68, 95% CI: 6.01–23.35, *p* = 0.001).

Age‐stratified multivariable logistic regression analyses, adjusted for BMI, parity, and physical activity, were used to examine associations between AMH levels and metabolic outcomes, including T2DM, HTN, MetS, and dyslipidemia in the PCOS, healthy, and suspicious groups (Table 3).

**TABLE 3:** Logistic regression of AMH and its association with T2DM, HTN, MetS, and dyslipidemia by age.

Clinical outcome	Group	< 35	≥ 35
		OR (95% CI)	OR (95% CI)
T2DM	Healthy	0.92 (0.65–1.30)	**0.63 (0.41–0.97)**
	PCOS	0.81 (0.39–1.70)	0.70 (0.35–1.40)
	Suspicious	1.26 (0.87–1.83)	1.15 (0.80–1.68)
HTN	Healthy	1.04 (0.82–1.30)	1.02 (0.82–1.30)
	PCOS	1.60 (0.94–2.60)	0.81 (0.46–1.46)
	Suspicious	1.08 (0.80–1.50)	1.26 (0.88–1.80)
MetS	Healthy	1.00 (0.82–1.23)	0.96 (0.74–1.25)
	PCOS	**2.60 (1.20–6.20)**	1.40 (0.65–3.00)
	Suspicious	0.98 (0.72–1.30)	0.94 (0.62–1.50)
Dyslipidemia	Healthy	1.30 (0.87–2.00)	1.25 (0.65–2.40)
	PCOS	1.58 (0.56–4.50)	3.83 (0.2–7.10)
	Suspicious	0.75 (0.42–1.34)	0.30 (0.08–1.04)

*Note:* All models were adjusted for BMI, parity, and physical activity. Statistically significant associations (*P* < 0.05) are shown in bold.

Abbreviations: HTN, hypertension; MetS, metabolic syndrome; T2DM, type 2 diabetes mellitus.

In healthy women, AMH was associated only with T2DM: among those aged ≥ 35 years, higher AMH was linked to a 37% lower likelihood of T2DM (OR: 0.63, 95% CI: 0.41–0.97, *p* = 0.036). In women with PCOS aged < 35 years, higher AMH was associated with 2.6‐fold higher odds of MetS (OR: 2.6, 95% CI: 1.2–6.2, *p* = 0.02).

## 4 Discussion

This population‐based study, which included both unselected women with PCOS representing relatively milder phenotypes and healthy community controls, demonstrated a consistent age‐related decline in AMH levels across the healthy, PCOS, and suspicious groups. However, this decline was notably less pronounced among women with PCOS. In healthy women, age explained nearly 40% of the variation in AMH levels, whereas in women with PCOS it accounted for only about 20%, highlighting distinct age‐related patterns of AMH decline in PCOS. Our findings also indicate that the association between AMH and metabolic outcomes varies according to age and reproductive status. Among healthy women aged ≥ 35 years, higher AMH levels were linked to a reduced likelihood of T2DM, suggesting a potential protective metabolic role of preserved ovarian function in later reproductive life. In contrast, among women with PCOS aged < 35 years, higher AMH levels were associated with a greater risk of MetS, suggesting that increased follicular activity may coexist with adverse metabolic features in younger women with PCOS. In the suspicious group, AMH was positively associated with both FAI and PRL levels. This pattern may reflect a subclinical endocrine phenotype characterized by mild hyperprolactinemia and preserved ovarian reserve without overt androgen excess. Notably, the positive association between AMH and FAI observed in this group was not evident among women with PCOS. This finding suggests that AMH may be more closely linked to androgenic activity in women with isolated PCOS features, whereas in established PCOS, AMH appears to primarily reflect ovarian follicular activity rather than androgen production.

Several studies have investigated age‐related changes in serum AMH levels in women with PCOS compared with healthy controls, although most have been conducted in clinical settings [22, 23]. Overall, these studies have shown an age‐associated decline in AMH, with a steeper decrease in healthy women than in those with PCOS, although some findings have been inconsistent. For example, Ran et al. reported a similar age‐related decline in AMH among women under 30 years in both PCOS and healthy groups [24], whereas Hwang et al. observed no significant variation across age groups in women with PCOS [8]. Likewise, Robin et al. found that age strongly influenced AMH levels in healthy controls but was less useful for characterizing women with PCOS [25]. In this context, our population‐based analysis, minimizing the “disease severity” bias typical of clinical samples, confirmed a weaker age–AMH relationship in PCOS compared with healthy women. This attenuation may stem from PCOS‐related hormonal imbalances, a higher count of antral follicles, and greater AMH production per follicle [26], all of which can elevate AMH levels independently of age. In addition, lifestyle factors, including obesity and diet, genetic determinants of ovarian function, and the use of hormonal contraceptives or fertility treatments may further influence AMH concentrations independently of aging [27].

Notably, AMH levels in our cohort were lower than those reported in some clinic‐based studies, including among women with PCOS, likely reflecting our population‐based design, which captures milder phenotypes typically underrepresented in clinic‐based settings. The mean age of participants (mid‐30s) also corresponds to a stage of physiological AMH decline. Additionally, population‐based evidence indicates that Iranian women may have a lower age‐specific ovarian reserve, with higher rates of premature ovarian insufficiency and early menopause [28] compared with global estimates [29] and Western populations [30], suggesting earlier reproductive aging. Together, these factors may explain the comparatively lower AMH levels observed in our study, while differences in assay methodology and ethnicity may also contribute.

The relationship between AMH and BMI has been explored from a pathophysiological perspective, with several mechanisms proposed to explain the association between increased adiposity and lower ovarian reserve. Obesity‐related chronic low‐grade inflammation may impair ovarian function [31], while adipose‐derived factors can interfere with ovarian signalling pathways [32]. Leptin, a hormone primarily secreted by adipocytes, has also been shown to suppress AMH gene expression [33]. Consistent with these mechanisms, our analysis demonstrated a weak inverse association between AMH and BMI in both the healthy and suspicious groups. Whether a similar relationship exists in women with PCOS remains uncertain. Previous studies, many of which were conducted in clinical settings, have reported inconsistent findings [34]. Although some studies have found weak associations between AMH and BMI in women with PCOS [8, 35, 36], others, including larger retrospective studies, report no significant link [22]. In our analysis of the PCOS group, AMH levels tended to decrease with increasing BMI; however, this association did not reach statistical significance. These findings suggest that the hormonal and metabolic disturbances characteristic of PCOS may attenuate or obscure the relationship between AMH and adiposity. Furthermore, the progressive weakening of this association from healthy to suspicious to PCOS women supports the hypothesis that increasing endocrine and metabolic dysregulation modifies the relationship between AMH and BMI.

The interplay between AMH and other hormones, particularly gonadotropins and androgens, is complex and remains incompletely understood, especially in PCOS. In this syndrome, AMH levels are elevated due to an increased number of preantral and small antral follicles, each producing more AMH per follicle [37]. Excess AMH may stimulate gonadotropin‐releasing hormone (GnRH) activity, leading to increased LH secretion and contributing to menstrual irregularity, anovulation, and infertility [6]. Elevated LH may, in turn, enhance ovarian androgen production by theca cells, contributing to symptoms like hirsutism and acne and further stimulating the production of AMH [38]. However, the strength and direction of these hormonal interactions vary across studies, likely due to differences in PCOS phenotype, obesity status, and the degree of hyperandrogenism. Several investigations have examined these relationships using AMH quartiles. Robin et al., in a retrospective cross‐sectional study, reported that women with PCOS in the highest AMH quartile (Q4) exhibited higher TT, A4, and LH but lower FSH compared with those in the lowest quartile (Q1), whereas SHBG levels remained similar [25]. Similarly, Qu et al. found progressive increases in LH and TT across AMH quartiles in women with PCOS [36]. Conversely, Aalpona et al. observed no significant differences in TT, TSH, or PRL across AMH quartiles, though AMH was positively correlated with TT in Spearman analysis [23]. Other studies also documented positive associations between AMH and testosterone and TT [22, 35], FAI [37], and LH or the LH/FSH ratio [22, 35, 39], particularly in more severe PCOS phenotypes characterized by elevated AMH, FAI, and LH levels [34, 39, 40]. Conversely, Hwang et al. reported a negative association between AMH and free testosterone in obese PCOS women [8]. Most studies, however, found no significant association between AMH and FSH [35, 36]. In our population‐based analysis, AMH was not significantly associated with hormonal parameters after adjustment for age and BMI in either healthy women or those with PCOS. This finding may reflect the inclusion of a broader spectrum of women, including non‐obese individuals and fewer cases of biochemical hyperandrogenism than are typically seen in clinic‐based cohorts, where obesity and severe hyperandrogenism may influence hormonal associations. These findings highlight the importance of considering body composition and disease severity when interpreting AMH as a hormonal biomarker in PCOS.

Interestingly, our findings showed a strong positive association between AMH and PRL levels in the suspicious group, women who met only one PCOS diagnostic criterion, whereas no such association was observed in the healthy or PCOS groups, consistent with previous studies [23, 36]. This unique hormonal interaction may reflect the presence of women with sole anovulation due to subclinical hyperprolactinemia in this group. Although mildly elevated PRL levels in this context are often clinically silent, they can still interfere with the hypothalamic–pituitary–ovarian (HPO) axis and disrupt ovulation. Mechanistically, higher AMH levels may indicate a greater pool of developing follicles, leading to increased ovarian oestrogen production. Elevated oestrogen can, in turn, stimulate pituitary PRL secretion through positive feedback [41, 42]. The clinical relevance of this AMH–PRL relationship emerged exclusively in the suspicious group, underscoring the need for a more precise evaluation of prolactin levels in individuals with isolated symptoms. Such assessments could help identify subtle ovulatory dysfunction and optimize early interventions to preserve reproductive health.

Evaluating the metabolic profile, this study found no significant associations between AMH levels and FBS, TC, TG, HDL, or LDL across the healthy, PCOS, and suspicious groups. Similar findings were reported by Malhotra et al. [22] and Ou et al. [36], who found no associations between AMH and glucose or lipid indices, though isolated relationships with TT [22] and inverse associations with the homeostatic model assessment for insulin resistance (HOMA‐IR) and fasting insulin [36] have been observed. Evidence from other studies remains inconsistent: a cross‐sectional analysis of PCOS women [43] found no link between AMH and dyslipidemia, whereas a meta‐analysis reported positive correlations with fasting glucose and lipid markers [44]. Associations between AMH and insulin resistance are likewise heterogeneous, with some studies linking elevated AMH to higher HOMA‐IR in PCOS [35, 39], while others observed no significant relationship [36, 45]. Conversely, a large population‐based cohort demonstrated that lower age‐specific AMH predicted greater T2DM risk in women without PCOS [46]. Recent data further suggest that higher AMH may protect premenopausal women, particularly non‐PCOS women, against nonalcoholic fatty liver disease (NAFLD) and its severe form, nonalcoholic steatohepatitis (NASH) [47]. In our study, higher AMH levels were independently associated with a lower likelihood of T2DM among healthy women aged ≥ 35 years, even after age‐stratified adjustment, suggesting that AMH may reflect preserved insulin sensitivity during the late reproductive years. These findings support a possible translational role for AMH as a biomarker of metabolic health beyond reproduction.

The relationship between AMH and MetS appears similarly complex. PCOS is associated with more than a threefold increased risk of MetS, even after adjustment for BMI, highlighting obesity‐independent mechanisms [48]. While some studies have linked higher AMH levels in PCOS to a greater MetS prevalence [49], others, including Aalpona et al., found no differences across AMH quartiles [23]. In the present study, higher AMH levels were associated with a 2.6‐fold increased risk of MetS among those with PCOS aged < 35 years, independent of BMI, parity, and physical activity. Notably, this association was not observed among women aged ≥ 35 years. This pattern may reflect age‐dependent effects, with AMH contributing to MetS via obesity‐independent pathways in younger women, whereas its influence diminishes during later reproductive life, consistent with evidence suggesting lower cardiovascular risk among older women with PCOS [50, 51, 52].

Mechanistically, insulin resistance plays a central role in the pathophysiology of PCOS and may represent a key link between AMH and metabolic dysfunction [53]. Insulin resistance in PCOS leads to compensatory hyperinsulinemia, which directly stimulates ovarian androgen production and reduces hepatic sex hormone–binding globulin synthesis, thereby increasing circulating free androgen levels [54, 55]. It may also influence hypothalamic–pituitary activity, thereby increasing LH secretion and further enhancing ovarian androgen production. Elevated androgen levels may subsequently impair insulin sensitivity, contributing to a bidirectional interaction between metabolic and reproductive disturbances in PCOS [53]. Within this context, changes in AMH levels may partly reflect ovarian responses to insulin‐driven hyperandrogenism [4]. However, due to the lack of fasting insulin measurements, we could not directly assess insulin resistance using HOMA‐IR. Consequently, we were unable to determine whether insulin resistance mediates the divergent metabolic associations of AMH observed in younger women with PCOS and older healthy women. Future studies incorporating direct measures of insulin sensitivity are warranted to further clarify this mechanistic interplay.

Our study has several important strengths. First, the inclusion of the “suspicious” group, women with isolated PCOS features, enabled the identification of an intermediate phenotype. Their hormonal and metabolic profiles, distinct from those of women with PCOS yet partially resembling those of healthy controls, may represent a transitional stage, with progression to full PCOS potentially influenced by genetic factors, environmental exposures (e.g., stress, diet, and toxins), lifestyle habits, and socioeconomic conditions [56]. Investigating such intermediate phenotypes could facilitate earlier detection and targeted interventions. Second, age‐stratified multivariable logistic regression improved the accuracy of assessing associations between AMH and metabolic outcomes by reducing confounding related to biological aging and highlighting age‐dependent associations. Finally, the use of a population‐based sample of unselected women with PCOS and the application of the Rotterdam criteria strengthen the robustness, reliability, and generalizability of our findings across both mild and severe phenotypes.

Our study also had several limitations. First, the sample size was insufficient to fully explore associations between AMH and various parameters across different PCOS phenotypes. Second, although insulin resistance plays a central role in the pathophysiology of PCOS, contributing to hyperandrogenism, ovulatory dysfunction, and metabolic disturbances, we were unable to directly assess insulin resistance using HOMA‐IR due to the lack of fasting insulin measurements. Consequently, we could not evaluate the relationship between AMH and insulin resistance, which may have provided further mechanistic insight into the divergent metabolic associations of AMH observed in healthy women and women with PCOS. Third, although equilibrium dialysis and ultrafiltration are considered the gold‐standard methods for measuring free testosterone, their high cost and limited availability preclude their use in large population‐based studies. Instead, we used the FAI, which correlates strongly with free testosterone and provides a reliable alternative when advanced techniques are not feasible [57]. Finally, although the TLGS is a population‐based, multicenter study, the cohort consisted exclusively of Iranian women from a defined urban population. Given documented ethnic variations in AMH levels, PCOS presentation, and metabolic risk, the generalizability of our findings to other populations may be limited. Future multiethnic studies are warranted to confirm these associations across diverse populations.

## 5 Conclusion

In summary, our population‐based study indicated that age‐related declines in AMH were less pronounced in women with PCOS than in healthy controls. While AMH was not correlated with BMI in the PCOS group, weak negative correlations were observed in the healthy and suspicious groups. Interestingly, higher AMH levels may be protective against T2DM in older healthy women, whereas elevated AMH levels in younger women with PCOS were associated with an increased risk of metabolic syndrome. Integrating these findings highlights that AMH may serve as a biomarker of metabolic health beyond its reproductive role, with its clinical interpretation dependent on age, BMI, and reproductive status. Importantly, assessing AMH in the context of life‐course health management may help identify women at risk for metabolic complications, supporting earlier preventive strategies and more personalized care. Further studies are warranted to validate these findings in diverse populations and to explore the mechanisms linking AMH to metabolic risk.

## Author Contributions

**Fatemeh Mahboobifard:** funding acquisition, investigation, conceptualization, methodology, supervision, project administration, writing – review and editing, writing – original draft, visualization. **Maryam Mousavi:** methodology, formal analysis, data curation, validation, visualization, software. **Marzieh Saei Ghare Naz:** writing – review and editing, investigation, visualization, resources. **Fahimeh Ramezani Tehrani:** conceptualization, writing – original draft, writing – review and editing, investigation, methodology, project administration, supervision, visualization, validation, resources. **Masoumeh Jorjani:** visualization, writing – review and editing. **Maryam Farahmand:** writing – review and editing, visualization, investigation.

## Funding

This work was supported by the Research Department of the School of Medicine, Shahid Beheshti University of Medical Sciences [Grant No. 43005456].

## Ethics Statement

The study protocol was developed in compliance with the principles of the Declaration of Helsinki and was approved by the Ethics Committee of Shahid Beheshti University of Medical Sciences (Ethics Code: IR.SBMU.MSP.REC.1402.65).

## Consent

Informed consent was obtained from all individual participants included in the study.

## Conflicts of Interest

The authors declare no conflicts of interest.

## Data Availability

The data that support the findings of this study are available from the corresponding author upon reasonable request.
